# Evaluation of Study Engagement With an mHealth Intervention (THR1VE) to Treat Diabetes Distress in Teens With Type 1 Diabetes: Randomized Clinical Trial

**DOI:** 10.2196/47089

**Published:** 2023-10-05

**Authors:** Lauren LeStourgeon, Erin Bergner, Karishma Datye, Randi Streisand, Sarah Jaser

**Affiliations:** 1 Department of Pediatrics, Vanderbilt University Medical Center, Nashville, TN, United States; 2 Department of Pyschology and Behavioral Health, Children's National Hospital, Washington, DC, United States; 3 Department of Psychiatry and Behavioral Sciences, George Washington University School of Medicine, Washington, DC, United States

**Keywords:** type 1 diabetes mellitus, positive psychology, adolescents, parental positive messaging, mHealth, engagement, diabetes, distress, teens, chronic health conditions, sex, age, device, race, ethnicity, text, mobile health

## Abstract

**Background:**

Positive psychology interventions demonstrate improvements in diabetes self-management and quality of life among adults with chronic health conditions, but few interventions for adolescents use this approach.

**Objective:**

This study describes engagement with a positive psychology intervention delivered via automated SMS text messages aimed at treating diabetes distress and improving diabetes outcomes. In addition, demographic and clinical predictors of intervention engagement were examined.

**Methods:**

Adolescents with type 1 diabetes (ages 13-17 years) who reported at least moderate diabetes distress were randomized to receive either the education or positive affect + education intervention, comprising 8 weeks of automated SMS text messages. Engagement was assessed as the response to the SMS text messages. Adolescents completed satisfaction surveys 3 months post intervention, and a subset of participants from both intervention groups completed exit interviews.

**Results:**

Adolescents in both groups reported high levels of satisfaction with the study, with 95% (163/172) reporting that they would participate again. Engagement with the SMS text messages was high; on average, adolescents in the positive affect + education group responded to 92.5% of intervention messages, and their caregivers responded to 88.5% of messages. There were no significant differences in rates of engagement related to adolescents’ sex, age, device use, or race/ethnicity.

**Conclusions:**

A positive psychology intervention for adolescents delivered via automated SMS text messages was feasible and acceptable across genders, ages, and racial/ethnic groups, suggesting potential for wider dissemination.

## Introduction

Many adolescents with type 1 diabetes (T1D) experience high rates of diabetes distress, or the emotional burden of living with diabetes [1], and struggle with diabetes management; in a national sample, only 17% of youth ages 13-17 years were meeting glycemic targets [2]. Interventions targeting family processes and adolescents’ coping skills have demonstrated modest effects [3-5]. Recently, behavioral interventions have focused on promoting resilience in youth with T1D [6], reinforcing adolescents’ diabetes-related strengths [7], and promoting positive parent-child communication around diabetes management behaviors [6,8] to improve psychosocial and glycemic outcomes. A positive psychology approach, focused on inducing positive affect, is an innovative way to improve outcomes among this high-risk population.

While positive psychology interventions have successfully improved adherence and self-efficacy among adults with chronic health conditions [9], few studies have evaluated this approach in adolescents, and the studies with adults used phone calls to deliver the intervention. The THR1VE intervention used evidence-based components [10] to induce positive affect among adolescents, including self-affirmation, gratitude, and positive parent messages, based on the broaden-and-build hypothesis that increasing positive affect improves people’s capacity to cope with stress in adaptive ways [11]. Given established associations between coping and psychosocial outcomes [12], the THR1VE intervention is based on the premise that increasing positive affect will reduce adolescents’ diabetes distress and improve glycemic outcomes. In addition, THR1VE included a caregiver component (providing positive messages) because adolescents’ perceptions that parents blame them for glucose levels and worry too much about complications contribute to their diabetes distress [13]. Because this positive psychology approach is relatively novel for adolescents, it is important to evaluate engagement and satisfaction in this age group, and to determine whether SMS text messaging is a feasible method of inducing positive affect.

This study builds on pilot work demonstrating the feasibility and acceptability of a brief positive psychology intervention for adolescents with T1D [14] by expanding the study to two sites and delivering the intervention remotely via Zoom sessions and automated SMS text messages. The intervention is aimed at reducing diabetes distress and improving glycemic outcomes among adolescents with T1D. In the current analyses, we describe the feasibility of intervention delivery, participant satisfaction and experience with the study, and the rates of engagement with the SMS text messaging intervention (response rate to automated SMS text messages), and examine demographic (age, gender, and site) and clinical predictors (baseline hemoglobin A_1c_ and use of diabetes devices) of engagement. In addition, we explore adolescents’ and caregivers’ experiences participating in the study and their feedback about the intervention. These findings have implications for adapting similar interventions for other pediatric populations.

## Methods

### Ethics Approval

This study was approved by the Vanderbilt Institutional Review Board (IRB# 191245).

### Procedures

This study was a randomized clinical trial (NCT03845465), and details of the protocol are described elsewhere [15,16]. Adolescents were eligible if they were aged 13-17 years, diagnosed with T1D for at least 12 months, had a cellular phone, and reported at least moderate diabetes distress, with a score of ≥34 on the Problem Areas in Diabetes–Teen Version (PAID-T). A score of ≥34 was chosen to screen for adolescents with an indication of moderate diabetes distress while allowing for a higher positive rate to meet recruitment and enrollment goals. Data collection occurred at baseline, 3 months, 6 months, and 12 months corresponding with diabetes clinic visits. After completing baseline measures, adolescents were randomized to receive either the education or positive affect + education (PA + EDU) intervention. Adolescents in the PA + EDU group received SMS text messages 5 days per week for 8 weeks after enrollment. These messages included self-affirmation messages, gratitude messages, and “mood booster” messages, and every 14 days they received a small gift (US $5 Amazon e-gift card code). Mood booster messages were selected based on ratings by 40 adolescents with T1D of inspirational quotes and jokes, and we created separate pools of mood booster messages for younger (ages 13-14 years) or older (ages 15-17 years) adolescents. Caregivers of adolescents in the PA + EDU group received messages once per week, reminding them to praise their child and asking them to reply yes or no if they gave their child a positive message that week. The SMS text messages were tailored to be sent at each adolescent’s and caregiver’s preferred time, and the start of each exchange asked the participant to “reply to this message with any text.” Each week, a research assistant reviewed participants’ responses to messages sent the previous week to identify and address system or user problems. If a participant did not respond to any messages within their first week of the intervention, a research assistant reached out to the participant to confirm they had received messages the past week and troubleshoot as needed (eg, participant thought the message was spam or the incorrect phone number was entered into REDCap).

As a measure of intervention acceptability, we examined engagement with the SMS text messaging intervention. We defined engagement as any response to the first message in the exchange. We also explored differences in engagement related to participant demographics.

### Measures and Data Collection

As part of the survey administered at the 3-month data collection time point, adolescents completed a brief evaluation survey asking how helpful they found the program (1=not helpful, 2=a little helpful, 3=somewhat helpful, 4=pretty helpful, 5=very helpful). They were also asked if they would recommend THR1VE to their friends, if they would participate again, and if the time spent on the study was worth their time. We considered the intervention acceptable if at least 50% of adolescents provided positive ratings of their perceptions of the intervention. A positive rating was indicated by answering that they found the program at least “somewhat helpful” and responding “yes” to questions about if they would recommend the program, if it was worth their time, and if they would participate again. Finally, they were asked which educational topics were most and least helpful to them.

In addition, we conducted brief qualitative interviews via Zoom (Zoom Video Communications) using purposive sampling (with respect to age, gender, race and ethnicity, site, and diabetes device use). Interviews were optional, and participants who completed them received an additional US $10 gift card. Interviews were recorded and transcribed. Qualitative data were analyzed using a content analysis method [17]. Two trained research staff individually structurally coded transcripts using NVivo software (version 12; QSR International), assigning codes to each caregiver/adolescent statement to capture its meaning. The research staff double-coded 17% of transcripts to review any discrepancies between assigned codes. Once consensus was achieved, the interrater reliability was established using NVivo.

## Results

### Participants

Participants in the study included 198 adolescents (n=115, 58.1% female; mean age 15.3, SD 1.4 years; n=114, 58% non-Hispanic White) with T1D (mean T1D duration 76.4, SD 44.5 months; mean hemoglobin A_1c_ 9.1%, SD 2.1%) and their caregivers (n=169, 85.4% female). A subsample of 66 adolescents and 63 caregivers completed a brief qualitative interview assessing study experience and perceptions (Table 1).

**Table 1.** Descriptive statistics of study participants and a subsample of participants who completed a brief qualitative interview.

Characteristic		Full sample (N=198)	Completed interview (n=66)^a^
**Site, n (%)**			
	VUMC^b^	108 (54.5)	29 (43.9)
	CNMC^c^	90 (45.5)	37 (56.1)
**Intervention group, n (%)**			
	Education	99 (50.0)	37 (56.1)
	Positive affect + education	98 (49.5)	29 (43.9)
Adolescent age (years), mean (SD)		15.3 (1.4)	15.3 (1.3)
**Adolescent gender, n (%)**			
	Male	83 (41.9)	30 (45.5)
	Female	115 (58.1)	36 (54.5)
**Adolescent race, n (%)**			
	African American/Black	47 (23.7)	13 (19.7)
	American Indian or Alaska Native	2 (1.0)	1 (1.5)
	Asian	8 (4.0)	4 (6.1)
	Biracial	16 (8.1)	8 (12.1)
	White	123 (62.1)	40 (60.6)
	Other	2 (1.0)	0 (0.0)
**Adolescent ethnicity, n (%)**			
	Non-Hispanic or Latinx	189 (95.5)	65 (98.5)
	Hispanic or Latinx	9 (4.5)	1 (1.5)
**Treatment type, n (%)**			
	Insulin pump	116 (58.6)	38 (57.6)
	Injections	82 (41.4)	28 (42.4)
Uses a CGM^d^, n (%)		161 (81.3)	54 (81.8)
Diabetes duration (months), mean (SD)		76.1 (44.4)	74.6 (44.2)
Baseline hemoglobin A_1c_ (%), mean (SD)		9.1 (2.1)	8.9 (1.8)
**Caregiver gender, n (%)**			
	Male	28 (14.1)	8 (12.7)
	Female	169 (85.4)	55 (87.3)
**Caregiver education, n (%)**			
	High school or less	29 (14.6)	5 (7.9)
	Some college	48 (24.2)	15 (23.8)
	College graduate	120 (60.6)	43 (68.3)
**Annual household income (US $), n (%)**			
	<50,000	51 (25.8)	13 (19.7)
	50,000-89,999	47 (23.7)	9 (13.6)
	90,000-149,999	42 (21.2)	21 (31.8)
	>150,000	56 (28.3)	22 (33.3)
**Caregiver marital status, n (%)**			
	Married/partnered	150 (75.8)	48 (76.2)
	Single/divorced/widowed	48 (24.2)	15 (23.8)

a The interview was completed by 63 caregiver-adolescent dyads and 3 adolescents (without their caregivers). One adolescent’s audio recording failed, and the data were unusable.

b VUMC: Vanderbilt University Medical Center.

c CNMC: Children’s National Medical Center.

d CGM: continuous glucose monitor.

### Feasibility

#### Intervention Delivery

Over the active study period (33 months), during which participants received their 8 weeks of study SMS text messages, we identified some instances where messages were not sent or received as expected. For example, if participants did not add the study phone number to their contacts list, some phone carriers blocked the messages as spam. Additionally, there were occasional system-wide glitches that interfered with sent SMS text messages. Overall, approximately 145 messages (n=35 adolescents and n=36 parents) were affected by system errors (out of >4400 scheduled text messages), representing only 3% of messages sent over the duration of the study.

#### Participant Perceptions

In general, both adolescents and their caregivers described favorable experiences participating in the study. Caregivers frequently noted that participating in the study was easy and was not burdensome:

*It was easy. It actually wasn’t time-consuming. I think the biggest thing was that it didn’t put a lot of pressure on me to have to take out a lot of time*

[mother of teen girl]

Caregivers found that the study survey questions were thought-provoking and allowed them to reflect on their experiences:

*The questions I could definitely relate to. It’s good for, kind of like, introspection...it kind of makes you think about your situation a lot more and how you can change it or do better you know, how I can do better in helping my daughter be more independent managing her diabetes and maybe get less irritable with her, angry with her about it*

[mother of teen girl]

Similarly, adolescents also reported that their study experience was easy and nonintrusive:

*I liked that it wasn’t like too probey. It didn’t feel like I was just like a test subject, like the surveys weren’t really long or super deep, they weren’t bothersome or troublesome*

[17-year-old girl]

In addition, teens also appreciated the gift card compensation. Although less common, some caregivers and teens noted that surveys were sometimes long and time-consuming, and a few participants reported that questions were repetitive or unclear.

Barriers to participation were not commonly reported by either caregivers or teens. However, several caregivers acknowledged forgetting to respond to the SMS text messages at times and minor issues with their mobile phones or receiving SMS text messages. Several teens and caregivers also noted that life circumstances occasionally interfered with participation, such as work and school schedules. For example, when asked if anything made it difficult or got in the way of their participation in the study, a mother of a teen girl explained:

*Just life in general. Just being busy. I would get some of the messages and stuff and I would be in the midst of working or cooking dinner or whatever I was doing...I’m like I didn’t get back to that, I forgot about it*.

### Acceptability

#### Study Interest

Both parents and adolescents were generally interested in the study. When asked about their initial interest, one mother said:

*I felt that anything that I could do to help [my child], I’m on board for. And if it’s going to help another teenager as well, so really for my child and other children*.

A 15-year-old girl stated, “I was interested because I think people need to know more about the emotional effects that type 1 diabetes has on kids.” The most common reasons for study interest reported by teens and caregivers included wanting to learn about diabetes and its management, gift card compensation, and wanting to help other people with diabetes.

#### Engagement With Text Messages

**Figure 1.**
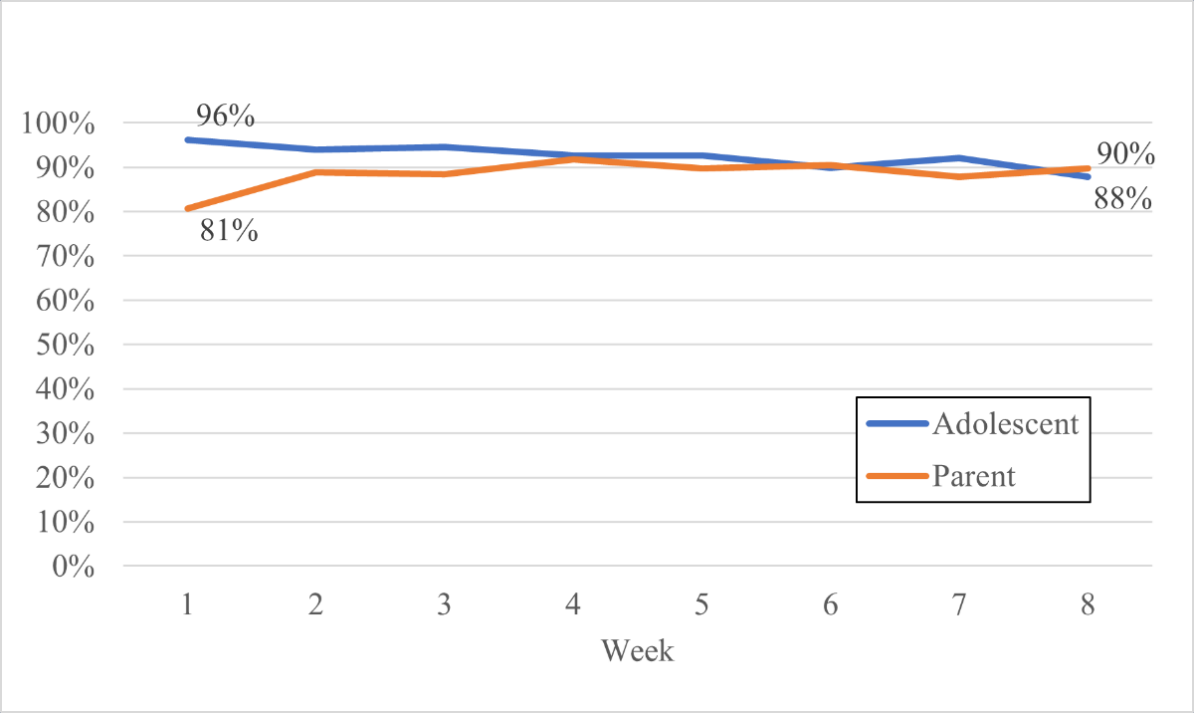
Average adolescent and parent response rate to automated texts by week.

The response rate to SMS text messages was high and remained fairly consistent across the 8-week intervention (Figure 1). Adolescents in the PA + EDU group responded to an average of 92.5% (SD 26.3%) of messages, and caregivers of adolescents responded to an average of 88.5% (SD 32%) of messages. We observed a small but significant decrease in the response rate over 8 weeks, with a high of 96.1% in week 1 and a low of 88.6% in week 8 among the adolescents (*F*_1,95_=4.27; *P*<.001); however, there was not a significant change in parents’ response over the 8 weeks (*F*_1,86_=0.99; *P*=.44).

When we examined the predictors of response rate, we found that the study site was the only significant factor; adolescents from the Vanderbilt University Medical Center site had significantly higher response rates at week 8 as compared to those from the Children’s National Medical Center site (no difference in the overall rate of response). Adolescent age, gender, and race/ethnicity were not significantly associated with response rate.

In qualitative interviews, teens in the EDU + PA group reported using the gratitude exercises or noticing things that made them happy, including thinking about family and friends, recent vacations, and activities they enjoy such as art and basketball. Teens reported the exercises were helpful and improved their mood. A 15-year-old girl said:

*I ride the bus home...I’d be in a grumpy mood and then I’d get a text and it would be asking about [what makes me happy] and so I’d say stuff about my dog. It just makes me happy to think about him sometimes*.

Similarly, a 17-year-old girl said:

*It was just a good perspective exercise where it just made me think about things more in a good way, not like in a sad way. I like that*.

The majority of teens reported continued use of positive affect exercises at the time of the interview. Caregivers also liked how the study SMS text messages engaged their children and that the study provided an outside source of encouragement and support for them. A mother of a teen girl said:

*What I liked was her getting text reminders with the positive feedback and kind of reminders and information...[My child] can also feel isolated, and this was like someone looking out for her besides just her family*.

Caregivers and teens in the PA + EDU group also reported positive experiences with using and receiving parental affirmations. Parents valued the teaching exercise and text reminders to give affirmations. A mother of a teen girl stated, “[The teaching exercise] made me really think about what I needed to—what to actually praise her for.” She went on to say how the exercise helped “separating diabetes from her and seeing her just as with diabetes...it’s been nice.” Another parent, a mother of a teen girl, appreciated the weekly reminders:

*I think it was just the right amount. You expected it every week, you know...it’s a good way to reflect on the week and actually wonder, “did I do something good?”*

Participants reported that parental affirmations were often oriented around academic achievements or family responsibilities.

*We had end-of- testing scores. I did really well on it so my mom was really proud of it*

[14-year-old boy]

Although parents received instructions to use affirmations unrelated to diabetes, several teens reported that their parents used diabetes-related affirmations with them, such as praise for checking their blood glucose levels.

Caregivers noted that they provided affirmations to teens face-to-face and through SMS text messages. More than half of caregivers in the EDU + PA group reported that they gave affirmations more frequently than once per week. Most parents described continued use of this strategy even after the weekly study reminders ended.

#### Study Satisfaction

AAdolescents were generally satisfied with the THR1VE program based on survey data completed by 86.9% (172/198) of adolescents, with 83.1% (n=143) reporting that the program was helpful in some way (somewhat helpful: n=65, 37.8%; pretty helpful: n=60, 34.9%; very helpful: n=17, 9.9%; average 3.34, SD 0.98), exceeding our benchmark of 50%. When asked if they would recommend the program to their friends, 87.2% (n=150) said yes, 94.8% (n=163) said that they would participate in it again, and 94.2% (n=162) said it was worth their time.

## Discussion

Feedback from participants indicated that a positive psychology intervention to induce positive affect delivered via tailored SMS text messages is feasible and acceptable for an adolescent population. The high rates of response to automated SMS text messages and lack of significant demographic predictors of engagement support that this approach was highly acceptable to adolescents with T1D. The response rate over the intervention period (8 weeks) demonstrates continued engagement, and adolescents and their parents reported continued use of positive affect exercises after the intervention ended.

Evaluating engagement in SMS text messaging interventions is necessary to understand whether this method is a viable option for health behavior change in adolescents. Given the relatively low rates of engagement with app-based interventions to improve diabetes management in youth, other approaches may be needed. For example, a randomized trial evaluating a diabetes management app for adolescents found that only 9% of participants had high engagement (using the app 3-7 days/week) [18], and a study evaluating a parent-developed app for diabetes management excluded 24% of participants from the analysis due to insufficient app use [19]. More recently, an app designed to facilitate positive parent-adolescent communication around diabetes management [8,20] found somewhat higher use; in a randomized pilot, the average app use was 58 out of 84 days for adolescents, but only 23 days for parents [8]. It is also unknown whether these apps would be acceptable for youth from minoritized racial and ethnic groups, as they were tested in predominantly non-Hispanic White youth or did not report on race or ethnicity. SMS text messaging may be a better way to reach adolescent populations, since even adolescents living in rural areas that have spotty Wi-Fi are likely to have access to cell phones [21], and teens report that SMS text messaging is their main source of communication across racial and ethnic groups [22].

The ideal “dose” of SMS text messaging is unclear, but adolescents in our study maintained high levels of response to messages 5 days per week. In interviews, the majority of teens in the EDU + PA group said that the amount of SMS text messaging was “just right,” a few teens said it was too many texts, and a few teens said it was too few texts. Most of the parents interviewed who received a weekly reminder, said it was a good amount, but several said they would like more frequent SMS text message reminders, such as a midweek text. By asking teens to report on their own sources of gratitude and personal attributes, we were able to personalize the messages without a lot of extra programming.

The inclusion of feedback from adolescents with T1D prior to starting the trial likely enhanced engagement; the problem we were addressing was meaningful for this population (diabetes distress), the mood booster SMS text messages (selected based on adolescents’ ratings) were appealing, and adolescents liked the amount/type of compensation (gift cards). Parents appreciated the reminders to give positive reinforcement/praise messages and that the intervention went beyond the tasks of diabetes management and addressed adolescents’ mood. Input from adolescents with T1D was essential in translating the positive psychology protocol developed for adults [9] to a younger population.

This study was limited by the involvement of adolescents with T1D who reported diabetes distress, so it may not be generalizable to other adolescent populations. While we had a relatively representative sample, it is possible that adolescents from different cultures may respond differently to positive psychology approaches, and future studies are needed to determine the amount of cultural tailoring needed to achieve high levels of engagement. Finally, it is challenging to evaluate acceptability versus user engagement for digital health interventions [23], and future work is needed to establish recognized benchmarks for acceptability.

Findings from this study support that a positive psychology intervention to induce positive affect delivered via automated SMS text messages is highly feasible and acceptable for adolescents and their caregivers. While the integration of feedback from the patient population is critical for a successful protocol, this approach may be translated to improve health outcomes in other pediatric populations.

## Supplementary material

10.2196/47089 Checklist 1 CONSORT-eHEALTH checklist (V 1.6.1).

## Abbreviations

PA + EDU: positive affect + education
PAID-T: Problem Areas in Diabetes–Teen Version
T1D: type 1 diabetes
